# Swimming with the Pigs: A Case of Severe Soft Tissue Infection during a Caribbean Vacation

**DOI:** 10.1155/2018/4092609

**Published:** 2018-10-24

**Authors:** Alexandra W. Dretler, Jesse T. Jacob, Nadine G. Rouphael

**Affiliations:** Division of Infectious Diseases, Department of Medicine, Emory University School of Medicine, Atlanta, Georgia, USA

## Abstract

A 74-year-old man presented to the emergency department with severe right leg cellulitis following a trip to the Bahamas where he swam in both chlorinated pools and the ocean. His blood cultures grew *Shewanella* species, a marine pathogen known to cause disease in humans, following exposure to seawater. He was treated with cefepime for a total of two weeks without needing any surgical intervention. The patient had complete resolution of infection and was able to return to his activities of daily living.

## 1. Case Presentation

**Figure 1:**
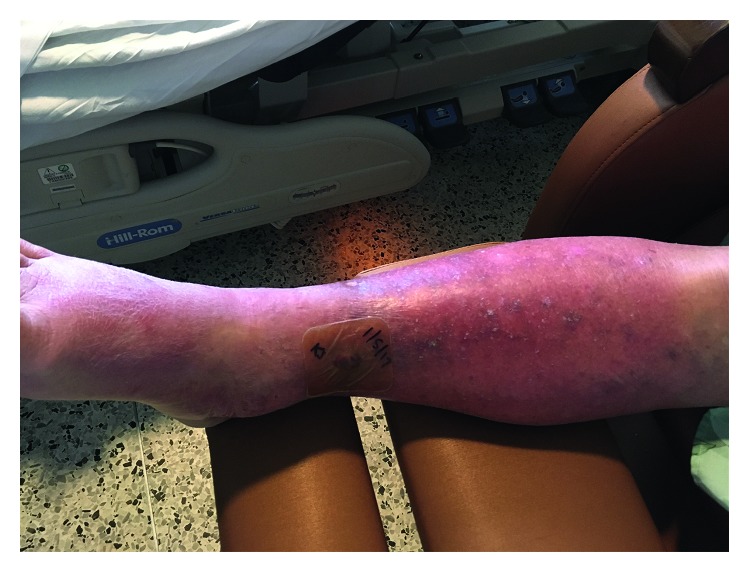
Image of the patient's leg.

A 74-year-old man presented to our tertiary care hospital's emergency department with severe pain and swelling in his right lower extremity. The patient had a skin biopsy on the right leg about 2 weeks prior to presentation for a possible skin cancer. He had no immediate complications following the biopsy, and the pathology was negative for cancer. One week after the biopsy, he went on a planned vacation with his extended family to the Bahamas. During the vacation, he went swimming in chlorinated pools at the resort and “swam with the pigs,” an adventure activity in the ocean with wild pigs. The night before his scheduled departure, he developed pain and redness at the site of his prior skin biopsy. Overnight, the pain became more severe, and he developed associated significant edema and worsening discoloration of the leg. On the morning of departure, he was unable to walk due to pain and also reported malaise and subjective fever and chills. The patient boarded a plane from the Bahamas to the US and went directly to the emergency department from the airport. On arrival, the patient had a temperature of 39.1 degrees Celsius, blood pressure of 109/55 mmHg, and his pulse was 140 bpm. His physical exam was notable for a circular wound on his right anterior shin with marked erythema, warmth, and induration surrounding, very tender to palpation (Figure 1). He had significant 2+ pitting edema extending up to his knee, and the erythema extended up in streaks onto his thigh and into his right groin, with enlarged inguinal lymph nodes.

The patient's past medical history was significant for coronary artery disease with a history of coronary artery bypass graft, mitral valve replacement with a porcine valve and graft, and atrial fibrillation. He was on warfarin chronically for anticoagulation and a beta blocker. He was a former smoker but quit over 40 years ago. He reported having 1 glass of wine daily and no known liver disease.

His creatinine was 1.49 mg/dL with normal liver function tests. Lactic acid level was elevated at 2.48 mmol/L. The white blood cell count was 10.6 × 10^3^/mcL with an absolute neutrophil count of 9.61 × 10^3^/mcL; hemoglobin and platelets were within normal limits. INR was 2.68 and PT was 31.5 seconds. Blood cultures were tested using the BACT/ALERT® (bioMérieux, Durham, NC) blood culture instrument. The aerobic and anaerobic bottles were both positive in this patient at 24 hours. The organism was an oxidase-positive, lactose nonfermenting gram-negative rod that produced mucoid, brownish colonies on sheep blood agar.

**Figure 2:**
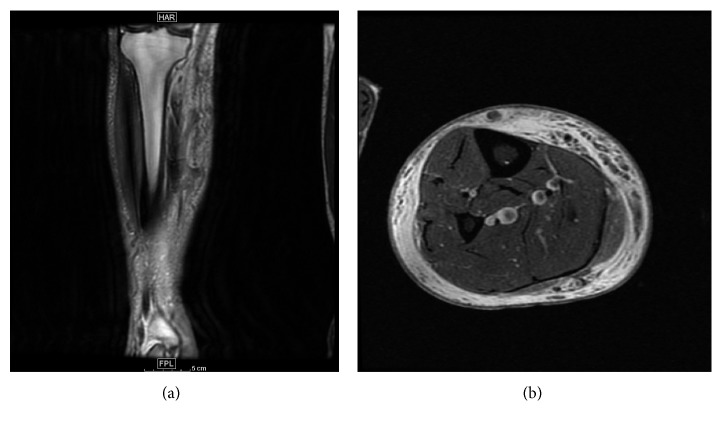
MRI images: (a) coronal T1 MRI Tibia/Fibula; (b) axial PD MRI.

Lower extremity venous duplex ultrasound of the right lower extremity did not reveal deep venous thromboembolism. MRI of the ankle and tibia/fibula with and without contrast was performed and revealed extensive cellulitis throughout the right lower extremity spanning the length of the knee to the ankle (Figure 2). There was no evidence of myositis, abscess, or osteomyelitis. Transthoracic echocardiogram was performed given the patient's history of porcine prosthetic mitral valve. No vegetations were seen.

The patient was started empirically on cefepime, doxycycline, and vancomycin to ensure adequate coverage of suspected marine pathogens. The oxidase-positive gram-negative rod growing in the patient's blood was ultimately identified as *Shewanella* species using the VITEK® MS matrix-assisted laser desorption ionization time-of-flight mass spectrometer (bioMérieux, Durham, NC) using IVD Knowledge Base Version 2.0 and Myla Version 4 information management software. The species was subsequently identified as *Shewanella putrifaciens* on the MicroScan WalkAway plus ID/AST system (Beckman Coulter, Brea, CA) using the Neg Breakpoint Combo 44 panel. The isolate was susceptible to cefepime, ceftazidime, piperacillin-tazobactam, and levofloxacin. MIC values were interpreted using CLSI breakpoints for other non-*Enterobacteriaceae*.

Cefepime was continued as monotherapy. Repeat blood cultures after initiation of antibiotics were negative. The swelling, erythema, and discoloration of the patient's right lower extremity gradually improved. No surgical intervention was required. Cefepime was continued as monotherapy for a total of 2 weeks since he had prolonged QTc.

## 2. Discussion

The differential for a patient presenting with a skin and soft tissue infection with both fresh and seawater exposure is broad. The most common marine pathogens to cause soft tissue infections include *Vibrio*, *Aeromonas*, *Shewanella*, *Plesiomonas*, *Pseudomonas*, *Mycobacterium*, *Clostridium*, and *Erysipelothrix*. *Vibrio, Aeromonas*, and *Shewanella* are all known to cause severe disease and have been associated with significant complications including bacteremia and necrotizing fasciitis. This patient ultimately was diagnosed with skin and soft tissue infection and concurrent bacteremia caused by *Shewanella* spp.

*Shewanella* spp. are found mainly in marine environments, but also in other forms of water, sewage, and many foods [1]. Predominantly found in Southeast Asia, Southern Europe, and South Africa, it has also been isolated in more temperate regions (including the Caribbean and the US), particularly in years with abnormally high recorded summer temperatures. The most common clinical syndrome associated with *Shewanella* infection is skin and soft tissue infection, but a variety of other syndromes have been described in the literature including bacteremia, hepatobiliary infection, bone and joint infection, central nervous system infection, ear and eye infections, endocarditis, and respiratory infection [1].

Risk factors associated with *Shewanella* infection include peripheral vascular disease and/or chronic open wounds and marine environment or seawater exposure [2]. Other associated comorbidities include liver disease, diabetes, and hypertension [3]. In a review of 27 cases of *Shewanella* skin and soft tissue infections [2], 22 out of 27 patients had limb involvement, mostly involving the lower extremities. Necrotizing fasciitis has been reported, but remains a rare complication of *Shewanella* infection [4, 5].

*Shewanella* spp. are often resistant to penicillin as well as first and second generation cephalosporin [6]. The majority of *Shewanella* isolates are susceptible to piperacillin-tazobactam, fluoroquinolones, third- and fourth-generation cephalosporins, aminoglycosides, and carbapenems [7]. Of note, the emergence of carbapenem resistance while on therapy with imipenem has been documented [8]. Most cases respond well to antibiotic therapy though surgical debridement may be needed.

*Shewanella* infections have been reported infrequently in the literature but represent an increasingly common cause of skin and soft tissue infection following exposure to seawater, especially as ocean temperatures continue to rise. Clinicians should be aware of this pathogen as a potential cause of infection, especially in those patients returning from trips to more temperate climates with seawater exposure. Empiric antibiotic therapy is often initiated before culture results are available. Empiric therapy should be tailored to appropriately cover the most common marine pathogens when a history of seawater exposure is elicited, including *Vibrio*, *Aeromonas*, and *Shewanella*. Most patients with infection due to *Shewanella* spp. have good outcomes with prompt initiation of appropriate treatment.
